# Long-Term Infectious Morbidity of Premature Infants: Is There a Critical Threshold?

**DOI:** 10.3390/jcm9093008

**Published:** 2020-09-18

**Authors:** Sharon Davidesko, Tamar Wainstock, Eyal Sheiner, Gali Pariente

**Affiliations:** 1 Department of Obstetrics and Gynecology, Soroka University Medical Center, Ben-Gurion University of the Negev, Beer-Sheva 84101, Israel; sheiner@bgu.ac.il (E.S.); galipa@bgu.ac.il (G.P.); 2 The Department of Public Health, Faculty of Health Sciences, Ben-Gurion University of the Negev, Beer-Sheva 84101, Israel; wainstoc@post.bgu.ac.il

**Keywords:** pregnancy, preterm birth, infectious, pediatric

## Abstract

In this study, we sought to ascertain a relationship between gestational age at birth and infectious morbidity of the offspring via population-based cohort analysis comparing the long-term incidence of infectious morbidity in infants born preterm and stratified by extremity of prematurity (extreme preterm birth: 24 + 0–27 + 6, very preterm birth: 28 + 0–31 + 6, moderate to late preterm birth: 32 + 0−36 + 6 weeks of gestation, and term deliveries). Infectious morbidity included hospitalizations involving a predefined set of International Classification of Diseases 9 (ICD9) codes, as recorded in hospital records. A Kaplan–Meier survival curve compared cumulative incidence of infectious-related morbidity. A Cox proportional hazards model controlled for confounders and time to event. The study included 220,594 patients: 125 (0.1%) extreme preterm births, 784 (0.4%) very preterm births, 13,323 (6.0%) moderate to late preterm births, and 206,362 term deliveries. Offspring born preterm had significantly more infection-related hospitalizations (18.4%, 19.8%, 14.9%, and 11.0% for the aforementioned stratification, respectively, *p* < 0.001). Multivariate analysis found being born very or late to moderate preterm was independently associated with long-term infectious morbidity (adjusted hazard ratio (aHR) 1.5, 95% confidence interval (CI) 1.27–1.77 and aHR 1.23, 95% CI 1.17–1.3, respectively, *p* < 0.001). A comparable risk of long-term infectious morbidity was found in the two groups of premature births prior to 32 weeks gestation. In our population, a cutoff from 32 weeks and below demarks a significant increase in the risk of long-term infectious morbidity of the offspring.

## 1. Introduction

Preterm birth refers to deliveries occurring prior to 37 completed weeks of gestation. Most preterm births are spontaneous, while up to 30% may be due to iatrogenic causes such as preeclampsia, fetal growth restriction, or multiple gestation [1]. The global incidence of preterm birth is reported to be between 5% and 18%, depending on location, with an average of 11% [2]. Annually, this translates to 15 million premature newborns, with this number increasing progressively [2]. Most (84%) of these are moderate to late preterm births, occurring between 32 + 0 and 36 + 6 weeks of gestation, with an additional 10% occurring between 28 + 0 and 31 + 6 weeks gestation, and the minority (5%) occurring prior to 28 weeks gestation [1]. Commonly recognized risk factors for preterm birth include previous preterm delivery [3], infectious or inflammatory disease [4], smoking [5], and multiple gestation [6]. It is well recognized that infectious disease explains at least partially the pathophysiology of premature onset of labor in the majority of cases as demonstrated by pathological evidence of chorioamnionitis in up to 75% of submitted placentas and positive membrane cultures in up to 60% of patients who delivered prematurely [7].

The risk of an offspring contracting an infectious disease during infancy or childhood is dependent on many factors including, but not limited to, exposure to infections via siblings or other children during daycare or school [8] and environmental exposures including smoking [9]. A link was also found between atopic disease and recurrent infections [10].

The increased morbidity and mortality rates of offspring born prematurely are often due to complications associated with the incomplete development of organ systems and the need for invasive care and life-support measures, which contribute to the pathogenesis of many complications including infections [11]. Of importance when searching for the causality in this association is the demonstrable suppression of numerous factors of the innate and acquired immune systems associated with an immature immune response with decreased efficacy [12]. There is evidence that this relationship between preterm delivery and infection is near linear, with the risk of infection increasing as gestational age decreases [13,14,15]. Commonly late preterm newborns are treated in the same way as full-term infants in clinical settings. Although late preterm infants are the least at risk for infectious complications when compared to more severely preterm infants, they also represent the majority of preterm infants and the fastest-growing subgroup [16], leading to a substantial burden of disease and highlighting the need for public health interventions to benefit this population of infants.

While much study has been devoted to the identification of the short-term consequences of preterm delivery and the development of interventions to limit morbidity and mortality, significantly less research has focused on the consequences extending beyond the neonatal and infant periods. One population-based study examined the association between gestational age and growth measurements with infection-related admissions to hospital throughout childhood [13], observing a 12% increase in infection-related hospital admissions for each decrease in gestational age below 39–40 weeks gestation, concluding that children born earlier have persistently increased rates of infection-related hospital admissions up to the age of 18 years. Our group previously published studies relating to the long-term risk of site-specific infections such as otitis media [17] and urinary tract infections [18] in relation to gestational age at delivery. All infer an inverse relationship between this risk and gestational age. This study set out to explore the relationship between preterm delivery, stratified according to severity, and the long-term incidence of all infectious morbidities of the offspring.

## 2. Methods

This retrospective population-based study included all singleton deliveries occurring between the years 1991 and 2014 at the Soroka University Medical Center (SUMC). SUMC is the largest birth center in the country and the sole tertiary hospital in the Negev area, serving a population of over 1.2 million inhabitants. The area has experienced positive immigration over the last two decades, with increasing annual birth rates from 10,000 to roughly 15,000 by the end of the study period. This study is based on nonselective population data.

Cases were divided into four groups according to the extremity of prematurity as defined by the WHO [19]—extreme preterm birth 24 + 0–27 + 6, very preterm birth 28 + 0–31 + 6, moderate to late preterm birth 32 + 0–36 + 6 weeks of gestation, and term deliveries of 37 or more completed weeks of gestation. Gestational age was determined according to the best obstetrical estimate determined by healthcare providers and used for clinical decision-making. As prenatal care in Israel is free, most patients complete a first-trimester ultrasound allowing correlation with the last menstrual period and accurate dating. If the ultrasound and last menstrual period were acceptably correlated, the last menstrual period was used for dating. If the last menstrual period was unknown or inconsistent with first-trimester biometry, the ultrasound data were used to determine gestational age.

Cases of multiple pregnancies and congenital malformations, which could be potentially confounding due to the relationship between both factors with preterm delivery, were excluded, as were cases of perinatal mortality (intrauterine fetal death, intrapartum death, and postpartum death). Outcomes included infection-related hospitalizations of all subtypes of the offspring up to the age of 18 years, excluding the infections occurring in the hospitalization immediately following delivery. Infectious morbidity was predefined in a set of ICD9 codes detailed in the Supplementary Materials, as recorded in hospital records.

Follow-up time was defined as time to event (infection-related hospitalization), and follow-up ended at first relevant hospitalization, or when censored for death of child (during hospitalization for unrelated morbidity), when the child reached 18 years of age (on the basis of date of birth), or at the end of the study period, whichever preceded.

Data were merged from two databases: the computerized hospitalization database of SUMC (“Demog-ICD9”) which includes demographic information and ICD-9 codes for all medical diagnoses made during hospitalization in any of the departments at SUMC including pediatric departments, and the computerized perinatal database of the obstetrics and gynecology department at SUMC, which consists of information recorded immediately following delivery by an obstetrician and reviewed by experienced medical secretaries for accuracy and completeness prior to entering it into the database. Coding is performed after assessing medical prenatal care records, as well as routine hospital documents.

### Statistical Analysis

Statistical analysis was performed using the SPSS package 23rd edition (IBM/SPSS, Chicago, IL, USA). Quantitative normally distributed variables were compared by analysis of variance (ANOVA), and categorical variables were compared using the chi-square test. Kaplan–Meier survival curves were used to compare cumulative infection-related hospitalization incidences over time according to gestational age at birth, divided into the four subgroups of gestational age as detailed above. The differences between the four cumulative morbidity curves (based on the different gestational age groups) were assessed using the log-rank test. A Cox proportional hazards model was used to establish an independent association between gestational age at birth and pediatric infection-related hospitalization risk while controlling for time to event, maternal age, birthweight, hypertensive disease, diabetes mellitus, and mode of delivery. Deliveries occurring at term (37 + 0 or more completed weeks of gestation) were considered as reference. All analyses were two-sided and a *p*-value <0.05 was considered statistically significant.

## 3. Results

During the study period 220,594 patients met the inclusion criteria, of which 125 (0.1%) were extreme preterm birth, 784 (0.4%) were very preterm birth, 13,323 (6.0%) were moderate to late preterm birth, and 206,362 were term deliveries. Table 1 summarizes the maternal characteristics and immediate perinatal outcomes according to the different gestational age groups. There was no difference in maternal age between the groups. Overall parity increased with increasing gestational age. The overall incidence of hypertensive disorders and cesarean deliveries decreased progressively with increasing gestational age. Diabetes was more common in women delivered preterm, although not for those who delivered extremely preterm, most likely due to these women delivering before the recommended gestational age for screening during pregnancy.

Offspring born preterm had significantly more hospitalizations due to infectious morbidity compared to term offspring (18.4%, 19.8%, 14.9%, and 11.0% for offspring born in extreme preterm birth, very preterm birth, moderate to late preterm birth, and term, respectively, *p* < 0.001, Table 2). Infectious morbidity was found to be increased in various organ systems and involving various pathogens as shown in Table 2. The most common infections in all gestational age groups were respiratory infections. The average age of the offspring at the time of infectious-related hospitalization increased with decreasing severity of prematurity as presented in Table 3 (*p* < 0.001).

A subanalysis of induced versus spontaneous deliveries (Table 4) shows that hospitalizations of offspring born extremely or very premature were more likely to occur following spontaneous delivery, whereas moderate to late preterm offspring and those born at term were more frequently hospitalized following induced delivery (*p* < 0.001).

Table 5 presents a Cox proportional hazards model, controlling for confounders including maternal age, birth weight, maternal diabetes, hypertensive disorders, mode of delivery, and year of birth. Being born very or moderate to late preterm was independently associated with long-term infectious morbidity (aHR 1.5, 95% CI 1.27–1.77 and aHR 1.23, 95% CI 1.17–1.3, respectively, *p* < 0.001). Extremely early preterm deliveries at 24 + 0–27 + 6 weeks were also found to increase the risk of long-term infectious morbidity (aHR 1.24, 95% CI 0.82–1.89, *p* = 0.304), but this association did not achieve statistical significance.

**Figure 1:**
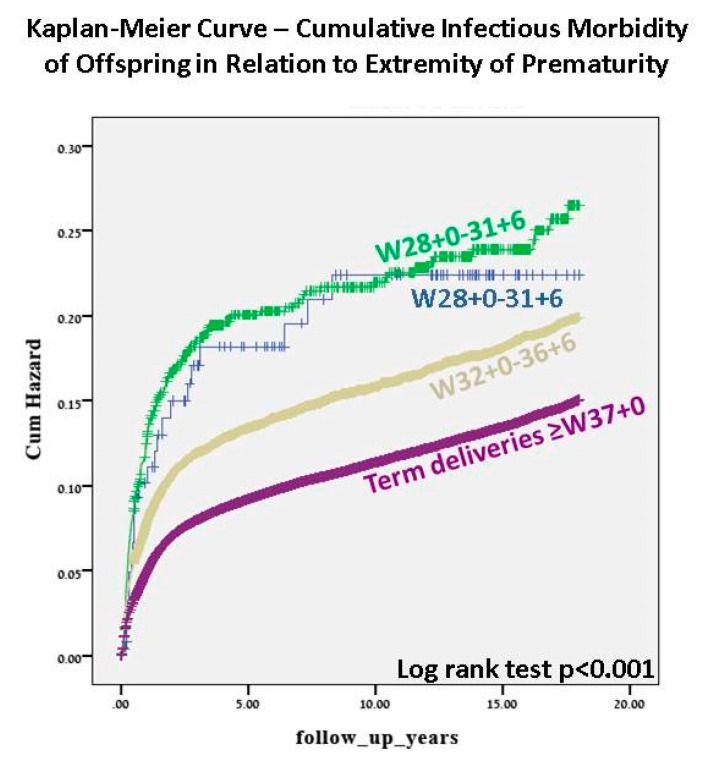
Kaplan–Meier survival curve—cumulative infectious morbidity of offspring in relation to extremity of prematurity.

**Figure 2:**
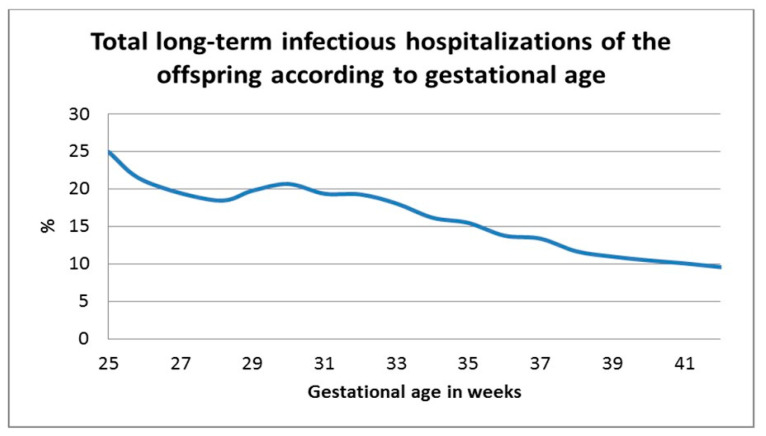
Total long-term infectious hospitalizations of the offspring according to gestational age.

The Kaplan–Meier survival curve (Figure 1) demonstrated progressively higher cumulative incidence of infectious morbidity with decreasing gestational age (log rank *p* < 0.001). The two groups with the earliest preterm deliveries showed a similar curve. Figure 2 demonstrates the univariate analysis of total infectious morbidity according to week of gestation at delivery. A general inverse relationship between gestational week at birth and infectious morbidity can be seen until term, with a further decrease in morbidity between early-term and full-term deliveries, although below 32 weeks, this relationship is less linear.

## 4. Discussion

Pediatric infections are a major cause of mortality and morbidity in both developed and developing countries, particularly in neonates and preterm babies with an estimated one million neonatal deaths annually worldwide attributed to infectious causes [20]. While infection has been studied in depth as a cause of preterm birth [4,7] and neonatal infection [20], data regarding the effect of preterm birth as a risk factor for long-term morbidity of the offspring beyond the neonate period is scarce [13]. Many factors affect an individual’s susceptibility to infection, including environmental exposures to smoking [9] and other children [8], in addition to the complex interaction of antenatal and intrapartum factors. As the focus of this study was the long-term impact of preterm delivery on the incidence of infectious-related hospitalization of the offspring in infancy and throughout childhood, we hoped to provide new insight into this important issue.

This large retrospective cohort study examined the long-term incidence of infectious-related hospitalizations stratified by extremity of prematurity and compared to offspring born at term. We showed that infectious morbidity is increasingly common with decreasing gestational age at birth, as shown in both the Kaplan–Meier survival curve and the Cox regression analysis model. From these models, it can be seen that, below 32 weeks gestation, there was no significant difference between the two groups with regard to the long-term infectious morbidity of the offspring; therefore, 32 weeks is the threshold below which the risk of morbidity significantly increases. Although this was not found to be statistically significant for extremely early preterm deliveries, Figure 2 demonstrates a general trend toward an inversely linear relationship between gestational age and long-term infectious morbidity of the offspring, possibly not reaching significance due to small numbers in this category. The relationship between gestational age and long-term infectious morbidity of the offspring was independent for confounding factors including maternal age, birthweight, maternal diabetes mellitus, hypertensive disorders, and mode of delivery. Despite changes in treatment over the decades of this study, the association between extremity of prematurity and long-term infectious-related hospitalization of the offspring remained significant after controlling for year of birth. This association was found to be significant for almost all the infectious diseases examined, including various pathogens and anatomic sites of infection including, but not limited to, common childhood infections of the respiratory and urinary systems, bacterial and viral infections, and more rare infections such as orthopedic infections and invasive bacterial infections (Table 2). The inclusion of these subgroups of infections allows us to make concrete conclusions that the hospitalization was due to infectious disease and not, for example, unexplained febrile illness. From our results, we can also see that, in addition to an increasing risk of infectious-related hospitalization during childhood with increasing severity of prematurity, the age at which the first hospitalization occurs is progressively earlier. This may reflect the growing maturity of the immune system throughout pregnancy [12], with children born with a more mature immune system at a later gestational age showing more resistance against infectious disease until later in childhood. A subanalysis of spontaneous versus induced deliveries revealed a significant association between labor induction and the incidence of infectious-related hospitalization. Induction at very young gestational ages (extremely and very preterm groups) is undergone for very few obstetric or maternal reasons, as the complications of prematurity in these cases can be severe for the offspring; therefore, as expected, in these groups more of the hospitalized offspring were born following spontaneous delivery. As gestational age increases into moderate and late preterm and term deliveries, the indications for induction are more numerous and induction for fetal indications is more common, possibly creating a cohort of offspring who are less healthy than their counterparts born following spontaneous onset of labor.

Among the possible confounders controlled for in the multivariate analysis, hypertensive disease was not found to be independently associated with long-term infectious morbidity of the offspring. Interestingly, in our study, the incidence of hypertensive disease decreased with increasing gestational age. While it is known from previous studies that the incidence of hypertensive diseases of pregnancy increases with increasing gestational age [21], this study examined deliveries and not ongoing pregnancies. An earlier onset of hypertensive disease in pregnancy likely results in increased severity of the disease and the occurrence of preterm delivery, whether medically indicated or spontaneous.

In our study, the rate of cesarean delivery increased significantly with decreasing gestational age. As shown in previous studies, newborns delivered via cesarean delivery are not exposed to the vaginal and perianal flora of the mother; rather, their initial exposure comprises nosocomial pathogens found in the operating room and dermal microbial colonization [22]. The offspring are likely to suffer from increased infectious-related hospitalizations throughout childhood [23]. This may be due to different immunologic function and response due to lack of exposure to the diverse array of antigens that the fetus is exposed to during vaginal delivery and the impact of this on the microbiota of the newborn as found in animal studies comparing the microbiome count and diversity according to mode of delivery in offspring as young as one month [24]. Breastfeeding failure and cessation are also more common following cesarean delivery, also contributing to the difference in flora, as breast milk contains bacteria important for immune function [22].

After controlling for the confounding effect of mode of delivery, decreasing gestational age at delivery was still significantly associated with increasing risk for long-term infectious morbidity of the offspring. The innate immune system, which provides the first line of defense against potential infection, develops and matures during fetal life and further during childhood [25,26]. It is known that both the innate and the acquired immune systems of the preterm newborn are compromised [12], increasing the susceptibility to infection and persisting throughout childhood. In addition, decreased neutrophil count and function, a lesser developed opsonization system, and an underdeveloped complement system with less effective signaling mechanisms between the innate and acquired immune systems are also characteristic of preterm neonates [27]. The preterm neonate has limited T helper 1 (Th1) response, which is critical for the development of immune memory and for fighting infection [12]. In addition, the premature infant is more likely to be exposed to complications associated with the incomplete development of organ systems and the need for invasive care, life-support measures, and total parenteral nutrition (TPN), which contribute to the pathogenesis of many complications including infections [11]. TPN in associated with changes in the microbiota, which is a known risk factor for infectious disease [28]. Interestingly, as shown in our study, the risk of long-term infectious morbidity continues to be increased in early-term offspring as compared to those delivered at full term, indicating that the disruption in immune mechanisms found in preterm infants may also exist, to a lesser extent, in these offspring delivered in early term. This group of infants was shown to be at risk for long-term morbidity including infectious urinary morbidity [18], otitis media [17], and respiratory morbidity [29] in previous studies from our group.

Perhaps due to this continuum of immune system maturity which continues throughout gestation and indeed throughout infancy, the univariate analysis in our study showed a general trend toward an inverse relationship between gestational age and long-term infectious morbidity. Beyond 32 weeks, this trend in clear, although, below this threshold, the relationship is less linear. This can also be seen in the Kaplan–Meier survival curves, which show a clear decrease from 32 weeks gestation in long-term infectious morbidity, but similar curves for earlier gestational ages.

The main limitation of this study is the retrospective design, with the analysis providing only evidence of association and not causation. Data regarding several potential confounding factors, such as trends in microorganisms and antimicrobial treatments over years were not available for further statistical analysis. An additional limitation is the inclusion of only hospital-related infectious morbidity, which likely includes only acute and severe infectious diseases, with no consideration of infectious morbidity diagnosed and treated in ambulatory settings; therefore, external validity of our findings is limited. As a single-center study, the results have limited generalizability compared to a multicenter study. However as SUMC is the largest birth center in the country and the sole tertiary hospital in the Negev area with little negative immigration, it is assumed that the majority of offspring would be followed up in our center, allowing true long-term follow-up. If immigration occurred, it can be assumed that the incidence was equal in all groups and, therefore, would not significantly impact the conclusions of this study. The large sample size is an additional strength of the study. Further studies to accurately identify the immune response in the preterm infant and studies to define the role of possible confounders such as breastfeeding, antibiotic use, and exposure to infections from siblings, amongst other maternal and pediatric characteristics, may help to clarify the relationship. In addition, more research is needed regarding interventions to improve the immune system function of these preterm infants and decrease the risk of long-term infectious morbidity. Several mechanisms have been proposed including probiotic supplementation [30] and maternal pre- and postnatal nutritional strategies [31].

## 5. Conclusions

In conclusion, a critical threshold of 32 weeks gestation was noted in our study, below which the risk of long-term infectious morbidity of the offspring was significantly increased. Interestingly, the risk of infectious diseases of the offspring in our study does not appear to be increased further below 28 weeks gestation. Despite this threshold, it can also be seen that each additional week of gestation further decreased the risk of long-term infectious morbidity, highlighting the need to consider the timing of medically indicated preterm delivery and even early-term delivery with regard to the long-term health of the offspring. Efforts should be made in clinical practice to prevent preterm delivery where possible and to optimize the timing of medically indicated induced deliveries as close to full term as medically possible without surpassing an unacceptable increase in the risk to both mother and fetus. Offspring born prematurely, specifically those who were born prior to 32 weeks of completed gestation, may benefit from increased surveillance during childhood for signs of infectious disease. Fulfilment of recommended vaccination programs, adherence to preventative strategies including general hygiene (for example, hand washing), and a high risk of suspicion allowing for early treatment of infectious disease may all contribute to reduce the number of children hospitalized due to infectious disease.

## Figures and Tables

**Table 1:** Demographic characteristics of pregnancies according to gestational age at delivery.

Characteristics		Extreme PTB (*n* = 125) (%)	Very PTB (*n* = 784) (%)	Moderate to Late PTB (*n* = 13,323) (%)	Term Births (*n* = 206,362) (%)	*p*-Value
Maternal Age		28.47	28.36	28.31	28.24	0.482
Parity	1	38.1	31.4	29.0	24.2	<0.001
	2–4	48.3	47.7	47.7	52.0	
	5+	13.6	20.9	23.3	23.8	
Mode of delivery	Vaginal	48.3	46.5	67.3	83.9	<0.001
	Assisted	0.0	0.6	1.7	3.3	
	Cesarean	51.7	52.8	31.0	12.7	
Birthweight (grams)		1096.13 ± 601.4	1644.43 ± 633.4	2540.32 ± 495.3	3270.5 ± 455.6	<0.001
Maternal diabetes		0.0	6.2	8.1	5.2	<0.001
Hypertensive disorders		8.5	19.1	12.9	4.7	<0.001

PTB, preterm birth.

**Table 2:** Long-term infectious morbidities in children (up to 18 years) according to gestational age at delivery.

Infectious Morbidity	Gestational Age at Delivery (Weeks)				*p*-Value
	24 + 0–27 + 6 (*n* = 125) *N* (%)	28 + 0–31 + 6 (*n* = 784) *N* (%)	32 + 0–36 + 6 (*n* = 13,323) *N* (%)	≥37 + 0 (*n* = 206,362) *N* (%)	
Bacterial infections	1 (0.8)	9 (1.1)	19 (0.1)	269 (0.1)	<0.001
Viral infections	4 (3.2)	14 (1.8)	151 (1.1)	1773 (0.9)	<0.001
ENT infections	4 (3.2)	20 (2.6)	281 (2.1)	3060 (1.5)	<0.001
GI infections	1 (0.8)	22 (2.8)	263 (2.0)	3448 (1.7)	0.004
Invasive bacterial infections	4 (3.2)	11 (1.4)	29 (0.2)	190 (0.1)	<0.001
Neonatal infections	2 (1.6)	5 (0.6)	55 (0.4)	549 (0.3)	<0.001
Ophthalmic infections	1 (0.8)	4 (0.5)	55 (0.4)	612 (0.3)	0.053
Orthopedic infections	0 (0.0)	5 (0.6)	25 (0.2)	315 (0.2)	0.005
Respiratory infections	10 (8.0)	70 (8.9)	1086 (8.2)	11,208 (5.4)	<0.001
Skin infections	4 (3.2)	6 (0.8)	122 (0.9)	1702 (0.8)	0.02
Urologic infections	2 (1.6)	15 (1.9)	144 (1.1)	1330 (0.6)	<0.001
Total infectious-related hospitalizations	23 (18.4)	155 (19.8)	1986 (14.9)	22,610 (11.0)	<0.001

ENT, ear, nose, and throat; GI, gastrointestinal.

**Table 3:** Average at of offspring at the time of first infectious-related hospitalization stratified by severity of prematurity at birth.

Gestational Age at Delivery (Weeks)	Age at Infectious-Related Hospitalization (Years)		*p*-Value
	Mean ± SD	Median	
24 + 0–27 + 6	1.852 ± 2.35	0.715	<0.001
28 + 0–31 + 6	2.088 ± 3.57	0.756	
32 + 0–36 + 6	2.812 ± 4.054	0.993	
≥37 + 0	3.317 ± 4.36	1.263	

**Table 4:** Incidence of infectious-related hospitalizations of induced versus spontaneous deliveries stratified by severity of prematurity.

Gestational Age (Weeks)	Spontaneous Labor *n* (%)	Induced Labor *n* (%)	*p*-Value
24 + 0–27 + 6	21 (18.6)	2 (16.7)	<0.001
28 + 0–31 + 6	142 (20.0)	13 (17.6)	
32 + 0–36 + 6	1521 (14.8)	465 (15.2)	
≥37 + 0	15,914 (10.6)	6696 (11.8)	
Total hospitalizations	17,598 (11.0)	7176 (12.0)	

The *p*-value is a result of both ANOVA and Kruskal–Wallis test.

**Table 5:** Cox regression model for infectious morbidity according to gestational age at delivery.

Gestational Age (Weeks)	Total		
	Adjusted HR	95% CI	*p*-Value
≥37 + 0	1		
32 + 0–36 + 6	1.27	1.206–1.335	<0.001
28 + 0–31 + 6	1.55	1.317–1.832	<0.001
24 + 0–27 + 6	1.48	0.976–2.231	0.065
Maternal age	0.987	0.984–0.989	<0.001
Birthweight	1	1.000–1.000	<0.001
Maternal diabetes	1.194	1.131–1.261	<0.001
Hypertensive disorders	1.044	0.988–1.103	0.128
Cesarean delivery	1.076	1.039–1.115	<0.001
Year of birth	1.107	1.105–1.110	<0.001

## Supplementary Materials

The following are available online at https://www.mdpi.com/2077-0383/9/9/3008/s1.
