# Supplementary material for: Long-Term Infectious Morbidity of Premature Infants: Is There a Critical Threshold?
Source: J Clin Med. 2020 Sep 18;9(9):3008. doi: 10.3390/jcm9093008 (PMC7563528; doi:10.3390/jcm9093008)
Supplement: Supplementary file 1 [file jcm-09-03008-s001.pdf]

Supplement Table

5901	ACUTE PYELONEPHRITIS
5950	ACUTE CYSTITIS
5959	CYSTITIS, UNSPECIFIED
5970	URETHRAL ABSCESS
5990	URINARY TRACT INFECTION, SITE NOT SPECIFIED
59010	AC.PYELONEPHRITIS WITHOUT LESION OF RENAL MEDULLARY NECROSIS
59080	PYELONEPHRITIS, UNSPECIFIED
59581	CYSTITIS CYSTICA
59589	OTHER SPECIFIED TYPES OF CYSTITIS
59780	URETHRITIS, UNSPECIFIED
59789	OTHER URETHRITIS
V1302	PERSONAL HISTORY OF URINARY (TRACT) INFECTION
008	INTESTINAL INFECTIONS DUE TO OTHER ORGANISMS
0030	SALMONELLA GASTROENTERITIS
0039	SALMONELLA INFECTION, UNSPECIFIED
0040	SHIGELLA DYSENTERIAE
0041	SHIGELLA FLEXNERI
0042	SHIGELLA BOYDII
0043	SHIGELLA SONNEI
0048	OTHER SPECIFIED SHIGELLA INFECTIONS
0049	SHIGELLOSIS, UNSPECIFIED
0051	BOTULISM
0059	FOOD POISONING, UNSPECIFIED
0068	AMEBIC INFECTION OF OTHER SITES
0069	AMEBIASIS, UNSPECIFIED
0070	BALANTIDIASIS
0071	GIARDIASIS
0078	OTHER SPECIFIED PROTOZOAL INTESTINAL DISEASES
0079	UNSPECIFIED PROTOZOAL INTESTINAL DISEASE
0084	INTESTINAL INFECTION DUE TO OTHER SPECIFIED BACTERIA
0085	BACTERIAL ENTERITIS, UNSPECIFIED

0088	INTESTINAL INFECTION DUE TO OTHER ORGANISM,NOT ELSEW.CLASS.
0090	INFECTIOUS COLITIS, ENTERITIS, & GASTROENTERITIS
0090	INFECTIOUS COLITIS, ENTERITIS, AND GASTROENTERITIS
0091	COLITIS,ENTERITIS,GASTROENTERITIS OF PRESUMED INF. ORIGIN
0092	INFECTIOUS DIARRHEA
129	INTESTINAL PARASITISM, UNSPECIFIED
00842	INTESTINAL INFEC. DUE TO PSEUDOMONAS
00843	INTESTINAL INFEC. DUE TO CAMPYLOBACTER
00845	INTESTINAL INFEC. DUE TO CLOSTRIDIUM DIFFICILE
00861	ENTERITIS DUE TO ROTAVIRUS
00862	ENTERITIS DUE TO ADENOVIRUS
00865	ENTERITIS DUE TO CALICIVIRUS
00867	ENTERITIS DUE TO ENTEROVIRUS, N.E.C.
00869	OTHER VIRAL ENTERITIS
1274	ENTEROBIASIS
1278	MIXED INTESTINAL HELMINTHIASIS
1279	INTESTINAL HELMINTHIASIS, UNSPECIFIED
1289	HELMINTH INFECTION, UNSPECIFIED
5902	RENAL AND PERINEPHRIC ABSCESS
V023	CARRIER OR SUSP.CARRIER OF OTHER GASTROINTESTINAL PATHOGENS
0022	PARATYPHOID FEVER B
0023	PARATYPHOID FEVER C
048	OTHER ENTEROVIRUS DISEASES OF CENTRAL NERVOUS SYSTEM
320	BACTERIAL MENINGITIS
322	MENINGITIS OF UNSPECIFIED CAUSE
325	PHLEBITIS AND THROMBOPHLEBITIS OF INTRACRANIAL VENOUS SINUSES
326	LATE EFFECTS OF INTRACRANIAL ABSCESS OR PYOGENIC INFECTION
0470	MENINGITIS DUE TO COXSACKIE VIRUS
0471	MENINGITIS DUE TO ECHO VIRUS
0478	OTHER SPECIFIED VIRAL MENINGITIS
0479	UNSPECIFIED VIRAL MENINGITIS
0491	NON-ARTHOPOD-BORNE MENINGITIS DUE TO ADENOVIRUS
0499	UNSP.NON-ARTHROPOD-BORNE VIRAL DIS.OF CENTRAL NERVOUS SYSTEM
0630	RUSSIAN SPRING-SUMMER (TAIGA) ENCEPHALITIS

3200	HEMOPHILUS MENINGITIS
3201	PNEUMOCOCCAL MENINGITIS
3202	STREPTOCOCCAL MENINGITIS
3203	STAPHYLOCOCCAL MENINGITIS
3207	MENINGITIS IN OTHER BACTERIAL DISEASES CLASSIFIED ELSEWHERE
3208	MENINGITIS DUE TO OTHER SPECIFIED BACTERIA
3209	MENINGITIS DUE TO UNSPECIFIED BACTERIUM
3229	MENINGITIS, UNSPECIFIED
3236	POSTINFECTIOUS ENCEPHALITIS
3238	OTHER CAUSES OF ENCEPHALITIS
3238	OTHER CAUSES OF ENCEPHALITIS, MYELITIS AND ENCEPHALOMYELITIS
3239	UNSPECIFIED CAUSE OF ENCEPHALITIS
3239	UNSPECIFIED CAUSE OF ENCEPHALITIS, MYELITIS,AND ENCEPHALOMYELITIS
3240	INTRACRANIAL ABSCESS
3241	INTRASPINAL ABSCESS
3249	INTRACRANIAL AND INTRASPINAL ABSCESS OF UNSPECIFIED SITE
06641	WEST NILE FEVER WITH ENCEPHALITIS
32082	MENINGITIS DUE TO GRAM-NEGATIVE
32089	MENINGITIS DUE TO OTHER SPECIFIED BACTERIA
32361	INFECTIOUS ACUTE DISSEMINATED ENCEPHALOMYELITIS (ADEM)
32381	OTHER CAUSES OF ENCEPHALITIS AND ENCEPHLOMYELITIS
32382	OTHER CAUSES OF MYELITIS
0031	SALMONELLA SEPTICEMIA
0038	OTHER SPECIFIED SALMONELLA INFECTIONS
00321	SALMONELLA MENINGITIS
00323	SALMONELLA ARTHRITIS
00329	OTHER LOCALIZED SALMONELLA INFECTIONS
04186	HELICOBACTER PYLORI (H. PYLORI) INFECTION
0119	UNSPECIFIED PULMONARY TUBERCULOSIS
01090	PRIM. TB. INFEC., UNSP.TYPE, UNSP.EXAMINATION
01120	TB. OF LUNG + CAVITATION, UNSP. EXAMINATION
01190	UNSP. PULMONARY TB., UNSP. EXAMINATION
01194	UNSP. PULMONARY TB., FOUND BY BACTERIAL CULTURE
01304	TB. MENINGITIS, FOUND BY BACTERIAL CULTURE

01311	TUBERCULOMA OF MENINGES, BACT/HISTOL. EXAM. NOT DONE
01394	UNSP. TB. OF C.N.S. FOUND BY BACTERIAL CULTURE
01404	TB. PERITONITIS, FOUND BY BACTERIAL CULTURE
01485	OTHER TB. INTESTINES, CONFIRMED HISTOLOGICALLY
V1201	PERSONAL HISTORY OF TUBERCULOSIS
022	ANTHRAX
0073	INTESTINAL TRICHOMONIASIS
0074	CRYPTOSPORIDIOSIS
0075	CYCLOSPORIASIS
0082	INTESTINAL INFEC. DUE TO AEROBACTER AEROGENES
0205	PNEUMONIC PLAGUE, UNSPECIFIED
0209	PLAGUE, UNSPECIFIED
0783	CAT-SCRATCH DISEASE
0810	MURINE (ENDEMIC) TYPHUS
0820	SPOTTED FEVERS
0830	Q FEVER
0838	OTHER SPECIFIED RICKETTSIOSES
0839	RICKETTSIOSIS, UNSPECIFIED
00841	INTESTINAL INFEC. DUE TO STAPHYLOCOCCUS
0846	MALARIA, UNSPECIFIED
0859	LEISHMANIASIS, UNSPECIFIED
0879	RELAPSING FEVER, UNSPECIFIED
0909	CONGENITAL SYPHILIS, UNSPECIFIED
0940	TABES DORSALIS
0980	GONOCOCCAL INFEC.,ACUTE, OF LOWER GENITOURINARY TRACT
1000	LEPTOSPIROSIS ICTEROHEMORRHAGICA
1160	BLASTOMYCOSIS
1173	ASPERGILLOSIS
1175	CRYPTOCOCCOSIS
1177	ZYGOMYCOSIS (PHYCOMYCOSIS OR MUCORMYCOSIS)
1179	OTHER AND UNSPECIFIED MYCOSES
1209	SCHISTOSOMIASIS, UNSPECIFIED
1225	ECHINOCOCCUS MULTILOCULARIS INFECTION OF LIVER
1228	ECHINOCOCCOSIS, UNSPECIFIED, OF LIVER

1229	ECHINOCOCCOSIS, OTHER AND UNSPECIFIED
1270	ASCARIASIS
1363	PNEUMOCYSTOSIS
1369	UNSP. INFECTIOUS & PARASITIC DISEASES
1369	UNSPECIFIED INFECTIOUS AND PARASITIC DISEASES
04183	OTHER CLOSTRIDIUM PERFRINGENS INFECTION
08881	LYME DISEASE (ERYTHEMA CHRONICUM MIGRANS)
08882	BABESIOSIS
09840	GONOCOCCAL CONJUNCTIVITIS (NEONATORUM)
09882	GONOCOCCAL MENINGITIS
09886	GONOCOCCAL PERITONITIS
0239	BRUCELLOSIS, UNSPECIFIED
0269	UNSPECIFIED RAT-BITE FEVER
0270	LISTERIOSIS
0312	DISSEMINATED DISEASE DUE TO OTHER MYCOBACTERIA
0319	UNSPECIFIED DISEASES DUE TO MYCOBACTERIA
03283	DIPHTHERITIC PERITONITIS
0418	OTHER SPEC.BACTERIAL INF;IN CONDIT.CLASS.ELSEWHERE,UNSP.SITE
0419	UNSP. BACTERIAL INFECTION, UNSP. SITE
0419	UNSPEC.BACTERIAL INF;IN CONDIT. CLASSIF.ELSEWHERE,UNSP.SITE
04189	OTHER SPECIFIED BACTERIA INFECTION
99762	INFECTION (CHRONIC) OF AMPUTATION STUMP
V091	INFECTION WITH MICROORGAN. RESISTANT TO CEPHALOSPORINS/B-LACTAM ANTIBIOTICS
V0980	INFECTION WITH MICROORGAN. RESISTANT TO OTHER SPEC. DRUGS, WITHOUT MENTION OF RESISTANCE TO MULTIPLE DRUGS
V0991	INFECTION WITH DRUG-RESISTANT MICROORGAN.,UNSPEC. DRUG RESISTANCE NOS, WITH MULTIPLE DRUG RESISTANCE
0330	WHOOPING COUGH DUE TO BORDETELLA PERTUSSIS (B. PERTUSSIS)
0331	WHOOPING COUGH DUE TO BORDETELLA PARAPERTUSSIS
0339	WHOOPING COUGH, UNSPECIFIED ORGANISM
034	STREPTOCOCCAL SORE THROAT AND SCARLET FEVER
035	ERYSIPELAS
0340	STREPTOCOCCAL SORE THROAT
0341	SCARLET FEVER
0410	STREPTOCOCCUS INFECT.IN CONDITION CLASSIF.ELSEWHERE;UNSP.SITE
0411	STAPHYLOCOCCUS INFECT.IN CONDIT.CLASSIF.ELSEWHERE,UNSP.SITE

0411	STAPHYLOCOCCUS INFECTION, UNSP. SITE
0412	PNEUMOCOCCUS INFECT.IN CONDITION CLASSIF.ELSEWHERE;UNSP.SITE
0412	PNEUMOCOCCUS INFECTION, UNSP. SITE
04100	STREPTOCOCCUS INFEC., UNSP.
04101	STREPTOCOCCUS INFEC., GROUP A
04101	STREPTOCOCCUS INFEC., GROUP A (ADDITIONAL CODE)
04102	STREPTOCOCCUS INFEC., GROUP B
04103	STREPTOCOCCUS INFEC., GROUP C
04104	STREPTOCOCCUS INFEC., GROUP D (ENTEROCOCCUS)
04105	STREPTOCOCCUS INFEC., GROUP G
04109	OTHER STREPTOCOCCUS INFEC.
04110	STAPHYLOCOCCUS INFECTION, UNSP.
04111	METHICILLIN SUSCEPTIBLE STAPHYLOCOCCUS AUREUS
04111	STAPHYLOCOCCUS AUREUS INFEC.
04111	STAPHYLOCOCCUS AUREUS INFEC. (ADDITIONAL CODE)
04112	METHICILLIN RESISTANT STAPHYLOCOCCUS AUREUS
04119	OTHER STAPHYLOCOCCUS INFECTION
V0252	CARRIER OR SUSP. CARRIER OF OTHER STREPTOCOCCUS
V0259	CARRIER/SUSP. CARRIER OF OTHER SPECIFIED BACTERIAL DIS.
0360	MENINGOCOCCAL MENINGITIS
0362	MENINGOCOCCEMIA
0369	MENINGOCOCCAL INFECTION, UNSPECIFIED
03682	MENINGOCOCCAL ARTHROPATHY
03689	OTHER SPECIFIED MENINGOCOCCAL INFECTIONS
0380	STREPTOCOCCAL SEPTICEMIA
0381	STAPHYLOCOCCAL SEPTICEMIA
0382	PNEUMOCOCCAL SEPTICEMIA
0383	SEPTICEMIA DUE TO ANAEROBES
0388	OTHER SPECIFIED SEPTICEMIAS
0389	UNSPECIFIED SEPTICEMIA
03810	STAPHYLOCOCCAL SPETICEMIA, UNSP.
03811	METHICILLIN SUSCEPTIBLE STAPHYLOCOCCUS AUREUS SEPTICEMIA
03811	STAPHYLOCOCCUS AUREUS SEPTICEMIA
03812	METHICILLIN RESISTANT STAPHYLOCOCCUS AUREUS SEPTICEMIA

03819	OTHER STAPHYLOCOCCAL SEPTICEMIA
03840	SEPTICEMIA DUE TO GRAM-NEGATIVE ORGANISM, UNSPECIFIED
03841	SEPTICEMIA DUE TO HEMOPHILUS INFLUENZAE (H. INFLUENZAE)
03842	SEPTICEMIA DUE TO ESCHERICHIA COLI (E. COLI)
03843	SEPTICEMIA DUE TO PSEUDOMONAS
03849	OTHER SEPTICEMIA DUE TO GRAM-NEGATIVE ORGANISMS
038491	KLEBSIELLA SEPTICEMIA
0388 1	CANDIDA SEPTICEMIA
0388 2	ENTEROCOCCUS SEPTICEMIA
0388 3	ACITINOBACTER SEPTICEMIA
0413	FRIEDLANDER'S BACILLUS, UNSP. SITE
0413	FRIEDLANDER'S BACILLUS;CONDITION CLASSIF.ELSEWHERE,UNSP.SITE
0413	KLEBSIELLA PNEUMONIAE
0414	ESCHERICHIA COLI (E. COLI), UNSP. SITE
0414	ESCHERICHIA COLI (E. COLI), UNSP. SITE (ADDITIONAL CODE)
0414	ESCHERICHIA COLI(E. COLI),CONDITI.CLASSIF.ELSEWHERE,UNSP.SITE
0415	HEMOPHILUS INFLUENZAE IN CONDIT.CLASSIF.ELSEWHERE,UNSP.SITE
0415	HEMOPHILUS INFLUENZAE, UNSP. SITE (H.INFLUENZAE)
0416	PROTEUS (MIRABILIS,MORGANII), UNSP. SITE
0416	PROTEUS(MIRABILIS,MORGANII)CONDIT,CLASSIF.ELSEWHERE,UNSP.SITE
0417	PSEUDOMONAS INFEC., UNSP. SITE
0417	PSEUDOMONAS INFECT.IN CONDIT.CLASSIF.ELSEWHERE,UNSPEC.SITE
04185	OTHER GRAM-NEGATIVE ORGANISMS INFECTION
042	HUMAN IMMUNODEFIC. VIRUS (HIV) DIS. /AIDS
V08	ASYMPTOMATIC H.I.V INFECTION STATUS
0521	VARICELLA (HEMORRHAGIC) PNEUMONITIS
0527	CHICKENPOX WITH OTHER SPECIFIED COMPLICATIONS
0528	CHICKENPOX WITH UNSPECIFIED COMPLICATION
0529	VARICELLA WITHOUT MENTION OF COMPLICATION
0539	HERPES ZOSTER WITHOUT MENTION OF COMPLICATION
0540	ECZEMA HERPETICUM
0542	HERPETIC GINGIVOSTOMATITIS
0543	HERPETIC MENINGOENCEPHALITIS
0546	HERPETIC WHITLOW

0549	HERPES SIMPLEX WITHOUT MENTION OF COMPLICATION
0559	MEASLES WITHOUT MENTION OF COMPLICATION
0569	RUBELLA WITHOUT MENTION OF COMPLICATION
0570	ERYTHEMA INFECTIOSUM (FIFTH DISEASE)
0578	OTHER SPECIFIED VIRAL EXANTHEMATA
0579	VIRAL EXANTHEM, UNSPECIFIED
0723	MUMPS PANCREATITIS
0729	MUMPS WITHOUT MENTION OF COMPLICATION
0740	HERPANGINA
0743	HAND, FOOT, AND MOUTH DISEASE
05311	GENICULATE HERPES ZOSTER
05319	HERPES ZOSTER + OTHER NERVOUS SYSTEM COMPLICATIONS
05320	HERPES ZOSTER DERMATITIS OF EYELID
05329	HERPES ZOSTER + OTHER OPHTHALMIC COMPLICATIONS
05410	GENITAL HERPES, UNSPECIFIED
05440	HERPES SIMPLEX + UNSP. OPHTHALMIC COMPLICATION
05441	HERPES SIMPLEX DERMATITIS OF EYELID
05443	HERPES SIMPLEX DISCIFORM KERATITIS
05449	HERPES SIMPLEX WITH OTHER OPHTHALMIC COMPLICATIONS
05472	HERPES SIMPLEX MENINGITIS
05479	HERPES SIMPLEX + OTHER SPEC. COMPLICATIONS
05810	ROSEOLA INFANTUM, UNSPECIFIED
05829	OTHER HUMAN HERPESVIRUS ENCEPHALITIS
0784	FOOT AND MOUTH DISEASE
0701	VIRAL HEPATITIS A WITHOUT HEPATIC COMA
0701	VIRAL HEPATITIS A WITHOUT MENTION OF HEPATIC COMA
0709	UNSP. VIRAL HEPATITIS WITHOUT HEPATIC COMA
0709	UNSPECIFIED VIRAL HEPATITIS WITHOUT MENTION OF HEPATIC COMA
07030	VIRAL HEPATITIS B WITHOUT HEPATIC COMA & HEPATITIS DELTA -92
07030	VIRAL HEPATITIS B WITHOUT HEPATIC COMA,AC/UNSP.WITHOUT HEP. DELTA
07032	VIRAL HEPATITS B WITHOUT HEPATIC COMA,CHR. WITHOUT HEPATITIS DELT
V0261	HEPATITIS B CARRIER
V0262	HEPATITIS C CARRIER
075	INFECTIOUS MONONUCLEOSIS

0785	CYTOMEGALIC INCLUSION DISEASE
0785	CYTOMEGALOVIRAL DISEASE
0773	OTHER ADENOVIRAL CONJUNCTIVITIS
0774	EPIDEMIC HEMORRHAGIC CONJUNCTIVITIS
0779	UNSPEC.DISEASES OF CONJUNCTIVA DUE TO VIRUSES AND CHLAMYDIAE
07799	UNSP. DIS. OF CONJUNCTIVA DUE TO VIRUSES
0780	MOLLUSCUM CONTAGIOSUM
0781	VIRAL WARTS
07810	VIRAL WARTS, UNSPECIFIED
07811	CONDYLOMA ACUMINATUM
07812	PLANTAR WART
07819	OTHER SPECIFIED VIRAL WARTS
0790	ADENOVIRUS INF.IN CONDITIONS CLASSIF.ELSEWHERE,UNSP.SITE
0790	ADENOVIRUS INFECTION, UNSP. SITE
0791	ECHO VIRUS INFECTION, UNSP. SITE
0792	COXSACKIE VIRUS INFECTION, UNSP. SITE
0793	RHINOVIRUS INFECTION, UNSP. SITE
0798	OTHER SPECIFIED VIRAL INFECTION CLASSIF.ELSEWHERE,UNSP.SITE
0799	UNSP. VIRAL & CHLANYDIAL INFECTION
0799	UNSP.VIRAL INFECT.IN CONDITIONS CLASSIF.ELSEWHERE,UNSP.SITE
07889	OTHER SPECIFIED DISEASES DUE TO VIRUSES
07889	OTHER SPECIFIED DISEASES DUE TO VIRUSES AND CHLAMYDIAE
07950	RETROVIRUS, UNSP.,UNSP. SITE
07959	OTHER SPECIFIED RETROVIRUS
07989	OTHER SPEC. VIRAL INFECTION
07999	UNSP. VIRAL INFECTION
07999	UNSP. VIRAL INFECTION (ADDITIONAL CODE)
0796	RESPIRATORY SYNCYTIAL VIRUS (RSV)
1100	DERMATOPHYTOSIS OF SCALP AND BEARD
1101	DERMATOPHYTOSIS OF NAIL
1103	DERMATOPHYTOSIS OF GROIN AND PERIANAL AREA
1104	DERMATOPHYTOSIS OF FOOT
1105	DERMATOPHYTOSIS OF THE BODY
1109	DERMATOPHYTOSIS OF UNSPECIFIED SITE

1110	PITYRIASIS VERSICOLOR
1118	OTHER SPECIFIED DERMATOMYCOSES
1119	DERMATOMYCOSIS, UNSPECIFIED
1120	CANDIDIASIS OF MOUTH
1121	CANDIDIASIS OF VULVA AND VAGINA
1122	CANDIDIASIS OF OTHER UROGENITAL SITES
1123	CANDIDIASIS OF SKIN AND NAILS
1124	CANDIDIASIS OF LUNG
1129	CANDIDIASIS OF UNSPECIFIED SITE
11281	CANDIDAL ENDOCARDITIS
11282	CANDIDAL OTITIS EXTERNA
11284	CANDIDAL ESOPHAGITIS
11289	OTHER CANDIDIASIS OF OTHER SPECIFIED SITES
382	SUPPURATIVE AND UNSPECIFIED OTITIS MEDIA
3813	OTHER AND UNSPECIFIED CHRONIC NONSUPPURATIVE OTITIS MEDIA
3814	NONSUPPURATIVE OTITIS MEDIA, NOT SPECIFIED AS ACUTE OR CHRONIC
3819	UNSPECIFIED EUSTACHIAN TUBE DISORDER
3820	ACUTE SUPPURATIVE OTITIS MEDIA
3821	CHRONIC TUBOTYMPANIC SUPPURATIVE OTITIS MEDIA
3823	UNSPECIFIED CHRONIC SUPPURATIVE OTITIS MEDIA
3824	UNSPECIFIED SUPPURATIVE OTITIS MEDIA
3829	UNSPECIFIED OTITIS MEDIA
3831	CHRONIC MASTOIDITIS
3839	UNSPECIFIED MASTOIDITIS
3841	CHRONIC MYRINGITIS WITHOUT MENTION OF OTITIS MEDIA
3849	UNSPECIFIED DISORDER OF TYMPANIC MEMBRANE
38001	ACUTE PERICHONDRITIS OF PINNA
38002	CHRONIC PERICHONDRITIS OF PINNA
38003	CHONDRITIS OF PINNA
38010	INFECTIVE OTITIS EXTERNA, UNSPECIFIED
38015	CHRONIC MYCOTIC OTITIS EXTERNA
38022	OTHER ACUTE OTITIS EXTERNA
38023	OTHER CHRONIC OTITIS EXTERNA
38100	ACUTE NONSUPPURATIVE OTITIS MEDIA, UNSPECIFIED

38101	ACUTE SEROUS OTITIS MEDIA
38104	ACUTE ALLERGIC SEROUS OTITIS MEDIA
38110	CHRONIC SEROUS OTITIS MEDIA, SIMPLE OR UNSPECIFIED
38119	OTHER CHRONIC SEROUS OTITIS MEDIA
38129	OTHER CHRONIC MUCOID OTITIS MEDIA
38200	AC.SUPPURAT.OTITIS MEDIA WITHOUT SPONTAN.RUPTURE OF EARDRUM
38201	ACUTE SUPPURAT.OTITIS MEDIA WITH SPONTAN.RUPTURE OF EARDRUM
38300	ACUTE MASTOIDITIS WITHOUT COMPLICATIONS
38301	SUBPERIOSTEAL ABSCESS OF MASTOID
38330	POSTMASTOIDECTOMY COMPLICATION, UNSPECIFIED
38389	OTHER DISORDERS OF MASTOID
38400	ACUTE MYRINGITIS, UNSPECIFIED
38401	BULLOUS MYRINGITIS
38420	PERFORATION OF TYMPANIC MEMBRANE, UNSPECIFIED
38482	ATROPHIC NONFLACCID TYMPANIC MEMBRANE
38500	TYMPANOSCLEROSIS, UNSPECIFIED AS TO INVOLVEMENT
38510	ADHESIVE MIDDLE EAR DISEASE, UNSPECIFIED AS TO INVOLVEMENT
38530	CHOLESTEATOMA, UNSPECIFIED
38531	CHOLESTEATOMA OF ATTIC
38532	CHOLESTEATOMA OF MIDDLE EAR
38630	LABYRINTHITIS, UNSPECIFIED
38635	VIRAL LABYRINTHITIS
460	ACUTE NASOPHARYNGITIS (COMMON COLD)
462	ACUTE PHARYNGITIS
463	ACUTE TONSILLITIS
464	ACUTE LARYNGITIS AND TRACHEITIS
465	ACUTE UPPER RESPIRATORY INFECTIONS OF MULTIPLE OR UNSP.SITES
475	PERITONSILLAR ABSCESS
4610	ACUTE MAXILLARY SINUSITIS
4611	ACUTE FRONTAL SINUSITIS
4612	ACUTE ETHMOIDAL SINUSITIS
4613	ACUTE SPHENOIDAL SINUSITIS
4618	OTHER ACUTE SINUSITIS
4619	ACUTE SINUSITIS, UNSPECIFIED

4640	ACUTE LARYNGITIS
4644	CROUP
4650	ACUTE LARYNGOPHARYNGITIS
4658	ACUTE UPPER RESPIRATORY INFECTIONS OF OTHER MULTIPLE SITES
4659	ACUTE UPPER RESPIRATORY INFECTIONS OF UNSPECIFIED SITE
4720	CHRONIC RHINITIS
4730	CHRONIC MAXILLARY SINUSITIS
4731	CHRONIC FRONTAL SINUSITIS
4732	CHRONIC ETHMOIDAL SINUSITIS
4733	CHRONIC SPHENOIDAL SINUSITIS
4738	OTHER CHRONIC SINUSITIS
4739	UNSPECIFIED SINUSITIS (CHRONIC)
4741	HYPERTROPHY OF TONSILS AND ADENOIDS
4742	ADENOID VEGETATIONS
4748	OTHER CHRONIC DISEASE OF TONSILS AND ADENOIDS
4749	UNSPECIFIED CHRONIC DISEASE OF TONSILS AND ADENOIDS
4870	INFLUENZA WITH PNEUMONIA
4871	INFLUENZA WITH OTHER RESPIRATORY MANIFESTATIONS
4878	INFLUENZA WITH OTHER MANIFESTATIONS
4880	INFLUENZA DUE TO IDENTIFIED AVIAN INFLUENZA VIRUS
4881	INFLUENZA DUE TO IDENTIFIED NOVEL H1N1 INFLUENZA VIRUS
46400	ACUTE LARYNGITIS WITHOUT MENTION OF OBSTRUCTION
46410	ACUTE TRACHEITIS WITHOUT MENTION OF OBSTRUCTION
46420	ACUTE LARYNGOTRACHEITIS WITHOUT MENTION OF OBSTRUCTION
46430	ACUTE EPIGLOTTITIS WITHOUT MENTION OF OBSTRUCTION
46450	SUPRAGLOTTITIS WITHOUT MENTION OF OBSTRUCTION
47400	CHRONIC TONSILLITIS
47401	CHRONIC ADENOIDITIS
47410	HYPERTROPHY OF TONSIL WITH ADENOIDS
47411	HYPERTROPHY OF TONSILS ALONE
47412	HYPERTROPHY OF ADENOIDS ALONE
47822	PARAPHARYNGEAL ABSCESS
47824	RETROPHARYNGEAL ABSCESS
466	ACUTE BRONCHITIS AND BRONCHIOLITIS

480	VIRAL PNEUMONIA
490	BRONCHITIS, NOT SPECIFIED AS ACUTE OR CHRONIC
4660	ACUTE BRONCHITIS
4661	ACUTE BRONCHIOLITIS
4800	PNEUMONIA DUE TO ADENOVIRUS
4801	PNEUMONIA DUE TO RESPIRATORY SYNCYTIAL VIRUS
4802	PNEUMONIA DUE TO PARAINFLUENZA VIRUS
4808	PNEUMONIA DUE TO OTHER VIRUS NOT ELSEWHERE CLASSIFIED
4809	VIRAL PNEUMONIA, UNSPECIFIED
4841	PNEUMONIA IN CYTOMEGALIC INCLUSION DISEASE
4918	OTHER CHRONIC BRONCHITIS
46611	AC. BRONCHIOLITIS DUE TO RESPIRATORY SYNCYTIAL VIRUS (RSV)
46619	AC. BRONCHIOLITIS DUE TO OTHER INFECTIOUS ORGANISMS
49121	OBSTRUCTIVE CHR. BRONCHITIS WITH(ACUTE)EXACERBATION
V1261	PERSONAL HISTORY OF PNEUMONIA (RECURRENT)
481	PNEUMOCOCCAL PNEUMONIA
481	PNEUMOCOCCAL PNEUMONIA (STREPTOCOCUUS PNEUMONIAE PNEUMONIA)
483	PNEUMONIA DUE TO OTHER SPECIFIED ORGANISM
4820	PNEUMONIA DUE TO KLEBSIELLA PNEUMONIAE
4821	PNEUMONIA DUE TO PSEUDOMONAS
4822	PNEUMONIA DUE TO HEMOPHILUS INFLUENZAE (H. INFLUENZAE)
4823	PNEUMONIA DUE TO STREPTOCOCCUS
4829	BACTERIAL PNEUMONIA, UNSPECIFIED
4830	PNEUMONIA DUE TO MYCOPLASMA PNEUMONIAE
4831	PNEUMONIA DEU TO CHLAMYDIA
4838	PNEUMONIA DUE TO OTHER SPECIFIED ORGANISM
48230	PNEUMONIA DUE TO STREPTOCOCCUS, UNSPECIFIED
48231	PNEUMONIA DUE TO STREPTOCOCCUS, GROUP A
48241	PNEUMONIA DUE TO STAPHYLOCOCCUS AUREUS
99590	SYSTEMIC INFLAMMATORY RESPONSE SYNDROME, UNSPECIFIED
99591	SEPSIS
99591	SYSTEMIC INFLAMMATORY RESPONSE SYNDROME DUE TO INFECTIOUS PROCESS WITHOUT ORGAN DYSFUNCTION
99592	SEVERE SEPSIS
99592	SYSTEMIC INFLAMMATORY RESPONSE SYNDROME DUE TO INFECTIOUS PROCESS WITH ORGAN DYSFUNCTION

04181	OTHER MYCOPLASMA INFECTION
04181	OTHER MYCOPLASMA INFECTION (ADDITIONAL CODE)
101	VINCENT'S ANGINA
0400	GAS GANGRENE
0990	CHANCROID
0993	REITER'S DISEASE
0999	VENEREAL DISEASE, UNSPECIFIED
1236	HYMENOLEPIASIS
1307	TOXOPLASMOSIS OF OTHER SPECIFIED SITES
1309	TOXOPLASMOSIS, UNSPECIFIED
1320	PEDICULUS CAPITIS (HEAD LOUSE)
1322	PHTHIRUS PUBIS (PUBIC LOUSE)
1323	MIXED PEDICULOSIS INFESTATION
1329	PEDICULOSIS, UNSPECIFIED
1330	SCABIES
1340	MYIASIS
04082	TOXIC SHOCK SYNDROME
04089	OTHER SPECIFIED BACTERIAL DISEASES
04184	OTHER ANAEROBES INFECTION
05442	DENDRITIC KERATITIS
07988	OTHER SPEC. CHLAMYDIAL INFECTION
07998	UNSP. CHLAMYDIAL INFECTION
